# Therapeutic Potential of Probiotic-Derived P8 Protein as an Anti-Metastatic Agent in Colorectal Cancer

**DOI:** 10.3390/microorganisms13092175

**Published:** 2025-09-17

**Authors:** Byung Chull An, Seungwoo Kim, Jaewon Ha, Sang-Hyuk Seok, Jun Won Park, Yongku Ryu, Myung Jun Chung

**Affiliations:** 1 R&D Center, Cell Biotech, Co., Ltd., 50, Aegibong-ro 409 beon-gil, Gaegok-ri, Wolgot-myeon, Gimpo-si 10003, Republic of Korea; 2 Division of Biomedical Convergence, College of Biomedical Science, Kangwon National University, Kangwondaehak-gil, ChunCheon-si 24341, Republic of Korea

**Keywords:** therapeutic protein, anti-cancer target protein, P8-Smad1 protein-protein interaction, *Lactobacillus rhamnosus* KCTC 12202BP, anti-migration, anti-metastasis

## Abstract

We previously described the use of probiotics to deliver a *Lactobacillus rhamnosus*-derived therapeutic protein, P8, which has been identified as a candidate colorectal cancer (CRC) suppressor protein with anti-proliferation and anti-migration activities. P8 was found to penetrate cell membranes by endocytosis, suppressing cell proliferation through G_2_ cell cycle arrest. Despite the ability of P8 to suppress cell migration in vitro, its mechanism of action in CRC is unclear. We profiled the P8-interacting partner proteins using the pull-down method with His-tagged bait P8 and then identified them by LC-MS/MS. Among the interacting targets, we focused on the mothers against decapentaplegic homolog 1 (Smad1), which is well known as one of the important modulators of the bone morphogenetic protein (BMP)-derived migration pathway in CRC. The present study discovers that P8 prevents the phosphorylation of Smad1 or heterologous complexes within the Smad family, interfering with the importation of Smad1 or its complexes into the nucleus. Thus, P8 significantly inhibits the up-regulation of epithelial–mesenchymal transition (EMT)-related genes mediated by Smad1. P8 also inhibits the morphological changes required for cell migration or adhesion. P8 induces morphologic changes in DLD-1 cells, and their spheroid surfaces, resulting in a significant reduction of the number and length of filopodia, as well as the down-regulation of the expression of myosin X and its accumulation in filopodia tips. This phenomenon seems to be a major negative regulator of cell motility that could be of key importance in metastasis. Use of a mouse model of human CRC metastasis confirmed that P8 significantly suppresses the liver metastatic rate. Probiotic-derived protein P8 significantly suppresses CRC metastasis through inhibition of the Smad1-EMT signal pathway and cell–cell adhesion.

## 1. Introduction

Colorectal cancer (CRC) is a prevalent malignancy of the digestive tract and ranks fourth worldwide in cancer-related mortality [1,2]. Its incidence has continued to rise over recent decades despite prevention initiatives [3]. Metastatic dissemination remains the principal driver of therapeutic failure and death [1,4]. At diagnosis, approximately 20% of patients already present with stage IV disease, and 30–40% undergo treatment for relapses that are still considered potentially curable [5]. Metastasis proceeds through a multistep cascade in which malignant cells detach from the primary tumor, intravasate into blood or lymphatic vessels, circulate, extravasate, and ultimately colonize secondary organs [6]. The liver is most frequently involved, although spread to the lungs, bones, brain, and spinal cord is also observed. Over the course of illness, roughly one-fifth of patients develop distant metastases [7,8,9,10,11]. Overall, metastatic CRC remains difficult to cure, and durable remission is uncommon [12,13].

Although efforts have been made to cure CRC, mortality rates have been increasing for decades [1,2]. Traditional chemotherapy for CRC has disadvantages due to differences in the degrees of cell cytotoxicity and side effects that can damage healthy tissues, disadvantages that have a detrimental impact on patient quality of life. Agents with high selectivity and low cytotoxicity are needed to improve the prognosis and survival rate of patients with advanced cancer.

A novel approach to overcome the disadvantages of chemotherapy consists of screening for functional proteins with anti-CRC effects. Probiotics, including lactic acid bacteria, are human intestinal microbes generally regarded as safe and having a positive impact on human health. Probiotics are monitored systematically during the production of human and animal foods [14]. Food-grade bacteria are by definition safe to ingest. Accumulating studies of probiotics have reported that they suppress colorectal carcinogenesis by regulating multiple molecular pathways associated with tumor initiation and progression [15,16]. Several strains of the species *L. plantarum*, *L. rhamnosus*, *L. acidophilus*, *L. casei*, *Bifidobacterium longum*, *B. infantis*, *B. adolescentis*, and *B. breve* have been found to effectively suppress CRC [17]. Their action mechanisms may act by stimulating the host’s immune response and inhibition of cell proliferation, mediated through regulation of apoptosis, differentiation, and tyrosine kinase signaling [15].

Recent studies have focused on the development of novel biotherapeutic drugs based on probiotics. P8, a protein with anti-cancer activity against DLD-1 cells, has been isolated from *L. rhamnosus*. In a mouse colorectal cancer xenograft model, intraperitoneal administration of P8 resulted in a significant reduction in tumor mass, with decreases of up to 59% compared to controls [18]. Endogenously expressed P8 in DLD-1 cells had 2-fold greater anti-proliferative effects than exogenous P8. P8 was found to enter the cytosol of CRC cells by endocytosis and could infiltrate into the nucleus by an as yet unknown mechanism [18,19]. The anti-proliferative effect of P8 was attributed to G2 phase cell cycle arrest, which subsequently led to the downregulation of Cyclin B1/CDK1.

To determine the mechanism of action (MOA) by which P8 suppressed metastatic activity, proteins in DLD-1 cell lysates that bind to P8 were analyzed by the pull-down method [19,20,21], as were P8-interacting targets associated with anti-migration signaling. The effects of P8 on its targeted proteins, as well as on cell signaling pathways, cell morphology [22,23,24], and cell migration, were analyzed. In addition, the ability of P8 to act as an anti-metastatic agent in a mouse model of human CRC metastasis was determined. These findings, investigating the mechanism by which P8 down-regulated CRC metastasis, can provide insight into understanding the anti-cancer properties of probiotics and could help identify a novel biotherapeutic drug in probiotics [18,19,25,26,27,28]. Findings showing that P8 suppressed cell migration suggested that P8 might also have anti-metastatic activity against CRC [19].

## 2. Materials and Methods

### 2.1. Bacterial Strains and Culture

The P8 protein was derived from *Lactobacillus rhamnosus* (LR) KCTC 12202BP, originally isolated from human feces. The LR strain, obtained from the culture collection of Cell Biotech Co., Ltd. (Gimpo, Republic of Korea), was used as the probiotic source. *Escherichia coli* (*E. coli*) DH5α and BL21 (DE3) strains were purchased from Novagen (Madison, WI, USA) and cultured in Luria–Bertani broth (Difco; Fisher Scientific, Hampton, NH, USA) at 37 °C for 18–24 h.

### 2.2. Cell Culture

DLD-1, a human colorectal cancer cell line, was purchased from the Korean Cell Line Bank (KCLB; Seoul, Republic of Korea) and maintained in RPMI-1640 medium (Gibco, Grand Island, NY, USA) supplemented with 10% fetal bovine serum (Gibco) and 1% penicillin/streptomycin (Gibco) at 37 °C in a humidified atmosphere containing 5% CO_2_.

Matrigel on-top (MoT) culture was performed as previously reported [24]. For subsequent experiments, cells were cultured for 12 h to evaluate filopodia-like protrusion (FLP) formation, for 5 days to analyze adhesion and phosphorylation, or for 10 days to assess cell proliferation.

### 2.3. Construction and Purification of Recombinant His-Tagged P8 Protein from E. coli

Expression of recombinant P8 protein was achieved using the pET-28a vector containing an N-terminal 6 × His tag and TEV protease cleavage site. The construct was transformed into *E. coli* BL21 (DE3) (Novagen, Madison, WI, USA) and cultured in M9 medium to mid-logarithmic phase (OD_600_ ≈ 0.6). Protein overexpression was induced with 0.5 mM IPTG for selenomethionine (SeMet) incorporation and continued for 4 h. Cells were harvested, resuspended in 20 mM HEPES (pH 7.5) with 150 mM NaCl, lysed by sonication, and clarified by centrifugation. The supernatant was purified using Ni^2+^-NTA agarose (Qiagen, Valencia, CA, USA), washed with 20 mM imidazole, and eluted with 300 mM imidazole. The affinity tag was removed by TEV protease digestion in the presence of 1 mM DTT. Final purification and quality assessment of SeMet-P8 were performed by size exclusion chromatography on a HiLoad 26/60 Superdex 200 pg column (GE Healthcare, Chicago, IL, USA) equilibrated in HEPES buffer [18].

### 2.4. Known-Down of Smad1 in DLD-1 Cells

DLD-1 cells were seeded in 6-well plates at a density of 7 × 10^5^ cells per well and cultured overnight. Transfection was performed using Smad1 shRNA (Santa Cruz Biotechnology, Dallas, TX, USA) and Lipofectamine 3000 (Invitrogen, Paisley, UK) following the manufacturer’s recommendations [29]. Transfected cells were subsequently maintained in RPMI-1640 medium containing puromycin (Sigma-Aldrich, St. Louis, MO, USA) to establish stable knockdown lines.

### 2.5. Identification of P8-Interacting Partner Proteins from DLD-1 Lysate

Recombinant His-tagged P8 protein was overexpressed in *E. coli* BL21 (DE3) and purified using Ni^2+^-NTA affinity chromatography as previously reported [19]. To capture interacting partners, the P8–Ni^2+^-NTA complexes were incubated with soluble DLD-1 lysates for 24 h at 4 °C. Following incubation, unbound proteins were removed by multiple washes with PBS, and the retained complexes were eluted with 20 mM HEPES (pH 7.5), 150 mM NaCl, and 300 mM imidazole. Resin incubated without P8 was used as a negative control. The eluted proteins were separated on SDS-PAGE gradient gels, visualized by Coomassie Brilliant Blue staining, and subjected to in-gel trypsin digestion. Peptide fragments were analyzed by LC–MS/MS, and proteins were identified using the PS1 platform (NICEM Co., Ltd., Seoul, Republic of Korea) [19].

### 2.6. Co-Immunoprecipitation (Co-IP) Assays

To evaluate protein–protein interaction by immunoblotting, P8 was incubated with Smad1 (MyBioSource Inc., San Diego, CA, USA) in PBS for 24 h at 4 °C. Rabbit anti-P8 IgG was bound to protein G agarose beads, with excess anti-P8 IgG eluted from the protein G agarose beads with PBS. For immunoprecipitation, each mixture of P8 and Smad1 was applied to anti-P8 IgG immobilized protein G agarose beads; following incubation for 12 h at 4 °C, the unbound proteins were washed out. The beads were boiled in dodecyl sulfate (SDS)-containing sample buffer, followed by separation of the samples via SDS–polyacrylamide gel electrophoresis (PAGE), transfer to PVDF membranes, and subsequent immunoblotting as previously described.

### 2.7. Wound Healing Assays

DLD-1 cells (wild type and transfectants expressing control or Smad1 shRNA) were seeded in 6-well plates at a density of 5 × 10^6^ cells per well. After 24 h, a linear scratch was introduced at the center of each well using a pipette tip, followed by three washes with PBS. Cells were then cultured at 37 °C for 3 days, and wound closure was assessed daily under a microscope (Nikon, Tokyo, Japan).

### 2.8. MoT 3D Cell Culture, Staining, and Confocal Microscopy

The formation of filopodia and FLPs was examined using the 3D MoT culture method [30]. Briefly, 20 μL of Growth Factor-Reduced Matrigel (Corning, Glendale, AZ, USA) were dispensed into 96-well plates and allowed to polymerize for 45 min at 37 °C. Cells (2 × 10^4^) suspended in culture medium containing 2% Matrigel were then seeded in each well (final volume, 40 μL). After incubation for 4–7 days, cultures were fixed with 4% paraformaldehyde in medium for 10 min at 37 °C and permeabilized with 0.5% Triton X-100 in PBS on ice for 10 min. Non-specific binding was blocked for 1 h at room temperature using 0.1% bovine serum albumin, 0.2% Triton X-100, 0.05% Tween-20, and 10% goat serum (Sigma-Aldrich). For co-localization, cells were incubated with primary antibodies against myosin X and EpCAM diluted in blocking buffer for 1 h at room temperature, followed by fluorescently labeled secondary antibodies under the same conditions. Actin filaments were counterstained with Alexa Fluor™ 488–conjugated phalloidin (Invitrogen) for 30 min, and nuclei were mounted with ProLong Gold antifade reagent containing DAPI. Imaging was performed with an ImageXpress^®^ Micro Confocal microscope (Molecular Devices, Sunnyvale, CA, USA) using 4–60× objectives. Z-stack images encompassing the entire cell clone were acquired, and maximum intensity projections were generated.

### 2.9. Western Blot Analysis

DLD-1 cells were lysed in RIPA buffer supplemented with a protease inhibitor cocktail (Roche, Basel, Switzerland). Equal amounts of protein (40 μg) were resolved by SDS–PAGE and transferred onto PVDF membranes (Amersham Bioscience, Piscataway, NJ, USA). Membranes were blocked with 5% skim milk in T-TBS and incubated overnight at 4 °C with primary antibodies (1:1000; Cell Signaling Technology, Danvers, MA, USA). After three washes with T-TBS (15 min each), membranes were exposed for 1 h at 4 °C to HRP-conjugated secondary antibodies (Cell Signaling Technology) diluted in blocking solution. Glyceraldehyde 3-phosphate dehydrogenase (GAPDH) was used as an internal control. Protein bands were detected using an enhanced chemiluminescence kit (Millipore, Billerica, MA, USA), followed by autoradiography using a Chemi-doc™ Touch Imaging System (Bio-Rad Laboratories, Hercules, CA, USA).

### 2.10. Real-Time Quantitative RT-PCR

Quantitative real-time RT-PCR was performed according to established protocols [31], with primer sequences for each gene provided in Table S1.

### 2.11. Immunocytochemistry Using ImageXpress^®^ Micro Confocal Microscopy

DLD-1 cells were seeded on coverslips in 6-well plates and cultured for 24 h before treatment with P8 (0–40 μM) for an additional 72 h. Cells were then fixed with 3% paraformaldehyde for 15 min at room temperature, washed three times with PBS, permeabilized in 0.2% Triton X-100/PBS for 2 min, and rinsed again. To minimize nonspecific signals, samples were blocked with 4% BSA in PBS for 30 min. Cells were incubated overnight at 4 °C with a rabbit polyclonal anti-P8 antibody (Young In Frontier Co., Ltd., Seoul, Republic of Korea) or, alternatively, for 2 h at 4 °C with commercial antibodies from Cell Signaling Technology. After washing, protein detection was performed using FITC-conjugated goat anti-rabbit IgG (Jackson ImmunoResearch, West Grove, PA, USA) or Alexa Fluor 568–conjugated donkey anti-mouse IgG (Invitrogen). Nuclear staining was carried out with Hoechst 33258 (5 μg/mL; Sigma) for 1 h at room temperature, followed by PBS washes and mounting. Fluorescence images were acquired with an ImageXpress^®^ Micro Confocal microscope (Molecular Devices) and analyzed using MetaXpress Software v5.3.01 (Molecular Devices) [32].

### 2.12. Cell Proliferation Assay or ImageXpress Live/Dead

DLD-1 cells were seeded at a density of 1 × 10^3^ cells per well in 96-well plates and cultured at 37 °C for 24 h. P8 (0–40 μM) was then added, and incubation was continued for 72 h. Cell viability was assessed using the Cell Counting Kit-8 (Dojindo Laboratories, Tokyo, Japan) following the manufacturer’s instructions, and absorbance was measured with a SpectraMax M5 microplate reader (Molecular Devices, Sunnyvale, CA, USA). For staining, cells were fixed in 4% paraformaldehyde for 30 min and subsequently treated with crystal violet for 30 min before imaging.

### 2.13. Animals

All animals were maintained in a temperature-controlled facility (25 ± 1 °C) under a 12 h light/12 h dark cycle. Experimental procedures were approved by the Animal Research Committee of Kangwon National University (approval no. KW-220401-3, approval on 28 April 2022) and carried out in accordance with institutional and national guidelines for the care and use of laboratory animals. NOD-SCID-IL2rg^nul^ (NSG) immunodeficient mice were obtained from Jabio (Suwon-si, Republic of Korea).

### 2.14. Splenic Injection and Drug Treatment

Female NSG mice (7 weeks old) underwent allograft transplantation. For splenic implantation, mice were anesthetized with isoflurane, and 1 × 10^6^ DLD-1 cells suspended in 15 µL RPMI-1640 mixed with 15 µL Matrigel (356,231; BD Biosciences, San Jose, CA, USA) were injected into the exposed spleen. Treatments were initiated on the day of tumor inoculation and continued for 7 weeks: saline or P8 (10 mg/kg, intraperitoneally, four times weekly) and 5-FU (40 mg/kg, intraperitoneally, twice weekly). At the end of the treatment period, mice were sacrificed and necropsied.

### 2.15. Histopathology

Mouse liver lobes were fixed in 10% formalin, processed using standard protocols, and embedded in paraffin. Sections of 3 μm thickness were prepared and stained with hematoxylin and eosin (H&E). For immunohistochemistry (IHC), paraffin sections were dewaxed, rehydrated, and subjected to antigen retrieval by heating in 0.01 M citrate buffer (pH 6.0) at 100 °C for 20 min. Immunostaining was performed with ImmPRESS Peroxidase Polymer kits (Vector Laboratories, Burlingame, CA, USA) according to the manufacturer’s instructions. Slides were incubated with rabbit anti-P8 primary antibody, followed by peroxidase polymer–conjugated secondary antibodies for 30 min. Detection was carried out using ImmPACT DAB substrate (SK-4105; Vector Laboratories), and nuclei were counterstained with Meyer’s hematoxylin for 10 s. Negative controls were prepared by substituting the primary antibody with diluent.

To evaluate metastatic burden, H&E-stained liver sections were scanned with a Pannoramic SCAN slide scanner (3D HISTECH, Budapest, Hungary), and all lobes were analyzed for the number and size of metastatic foci using Case Viewer software (version 2.3; 3D HISTECH).

### 2.16. Statistical Analysis

Data are expressed as mean ± SD. Statistical significance was assessed by one-way ANOVA followed by Tukey’s post hoc test. Analyses were conducted using GraphPad Prism version 4 (GraphPad Software, La Jolla, CA, USA), and *p* values < 0.05 were considered significant.

## 3. Results

### 3.1. Anti-Cancer Properties of P8 Against DLD-1 Cells

**Figure 1:**
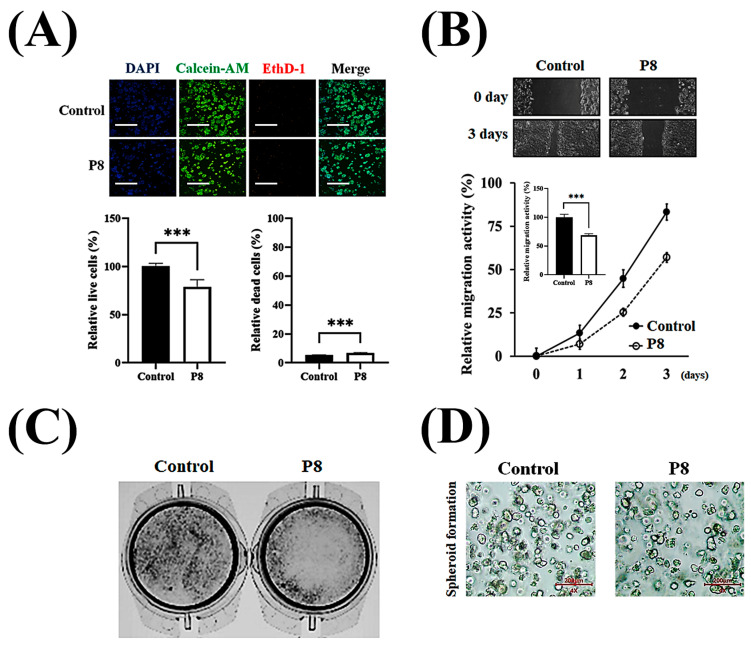
P8-derived anti-CRC properties in vitro. Characterization of the anti-CRC activity of P8 in vitro. (**A**) DLD-1 cells (3 × 10^3^/well) were treated with P8 (40 μM) for 72 h, and cell viability was assessed by live/dead staining (Syto9, green; EthD-1, red) and nuclear staining with Hoechst (blue), followed by imaging with ImageXpress^®^ Micro confocal microscopy (4× objective; scale bar = 200 μm). Data represent mean ± SEM from three independent experiments (*n* = 3). *** *p* < 0.001 vs. control. (**B**) Anti-migratory effects of P8 were evaluated by wound healing assays. DLD-1 cells (5 × 10^6^/well) were seeded in 6-well plates, scratched after 24 h, treated with P8 (40 μM), and cultured for 72 h. Wound closure was recorded daily under a light microscope (40× magnification) and quantified using ImageJ software (Version 1.54p). Data are mean ± SEM from three independent experiments (*n* = 3). *** *p* < 0.001 vs. Control. (**C**) Transwell assays assessing migration after 12 h of P8 treatment (40 μM). DLD-1 cells (1 × 10^6^/well) were seeded in 6-well plates, and migrated cells were visualized by crystal violet staining and examined microscopically (40× magnification). (**D**) Effect of P8 on spheroid formation in Matrigel 3D culture. DLD-1 cells (2 × 10^4^/well) were incubated with P8 (40 μM) for 8 days and imaged using ImageXpress^®^ Micro confocal microscopy (4× objective).

Investigation of the anti-cancer properties of P8 showed that P8 reduced the proliferation and migration of DLD-1 cells (Figure 1). P8 treatment reduced the growth of cancer cells by up to ~22% but did not alter the number of dead cells (Figure 1A). Previous studies reported that P8 arrests the cell cycle at G2 in DLD-1 cells through suppression of CDK1/Cyclin B1 expression, a critical regulator of this phase [18]. P8 also reduced cancer cell migration up to ~35% in a wound healing assay (Figure 1B), as well as reducing cell migration in a transwell assay (Figure 1C). Additionally, P8-treated spheroids were smaller and less spherical aggregations of cells than control spheroids (Figure 1D). These findings suggest that P8 might play a role in anti-metastatic activities, including cell proliferation and migration, as well as the maintenance of cell morphology and growth distribution.

### 3.2. Anti-Metastatic Target of P8 in DLD-1 Cells

**Figure 2:**
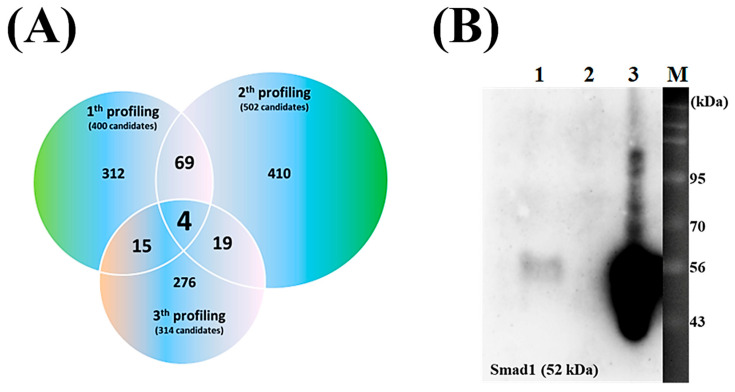
Characteristics of P8 target proteins in DLD-1 cells. (**A**) Venn diagram illustrating the overlap of P8-bound proteins identified in DLD-1 cell lysates across three independent experiments. Shown are the distributions in each experiment compared with the distributions of shared and specific P8 targets. The numbers in parentheses reflect the number of detectable targets. (**B**) Evaluation of P8 interaction with Smad1 by immunoprecipitation. Aliquots containing 1 μg each of P8 and Smad1 in PBS were mixed for 12 h and applied to an anti-P8 IgG-coupled protein A column; lane 1, unbound Smad1; lane 2, final wash fraction; lane 3, eluted fraction; lane M, size markers. Immunoprecipitation results were visualized using anti-Smad1 IgG.

In the recent P8 study, we have conducted a P8 target discovery experiment [19]. The experiment was designed to systematically separate proteins that interact or form complexes with P8, and His-tagged P8 and Ni^2+^-NTA resin were used to pull-down proteins interacting with P8, resulting in a complex of His-tagged P8 and Ni^2+^-NTA resin that bound proteins reacting with P8 in DLD-1 lysates that was successfully prepared. Consequentially, enriched P8-interacting targets were successfully obtained in elution fractions. Proteins interacting with P8 from three independent experiments were subjected to overlapping analysis, resulting in the isolation of four putative P8-interacting target proteins (Figure 2A, Table 1). The ability of these four candidate proteins to interact with P8 in vitro was assessed by co-IP assays. The detection of Smad1 in almost all eluted fractions indicated that Smad1 specifically interacted with P8 (Figure 2B, lane 3).

### 3.3. P8-Associated Anti-Migration and Anti-Adhesion Signaling Pathways in DLD-1 Cells

**Figure 3:**
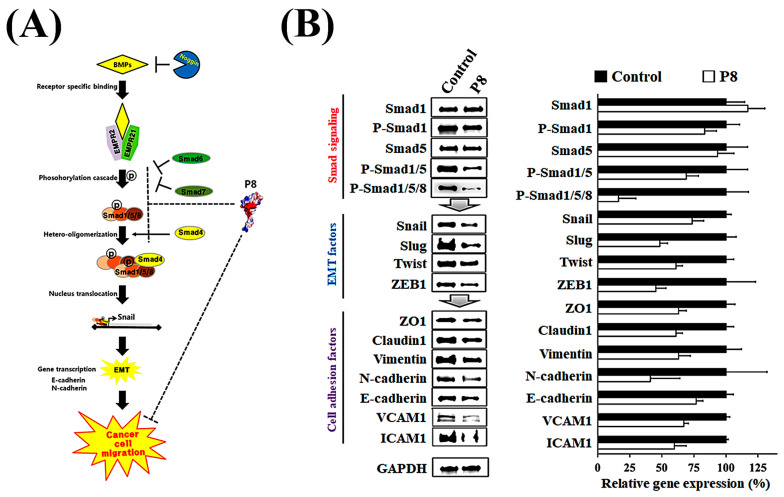
P8-associated anti-metastasis cell signaling in CRC. Inhibition of the BMP pathway strongly inhibited Smad1-induced EMT and cell adhesion in DLD-1 cells. (**A**) Schematic diagram of the BMP signal pathway with P8. After interacting with Smad1, P8 strongly interfered with Smad1-associated EMT signal transduction by inhibiting the phosphorylation of Smad1 and its complexes (Smad1/5 and Smad1/5/8). (**B**) Western blot analysis of whole-cell lysates from DLD-1 cells left untreated or treated with P8 (40 µM, 72 h). Protein identities are indicated on the left; GAPDH served as a loading control. Quantification of protein expression related to EMT and cell–cell adhesion. Band intensities were measured using ImageJ software (Version 1.54p) and normalized to GAPDH.

**Figure 4:**
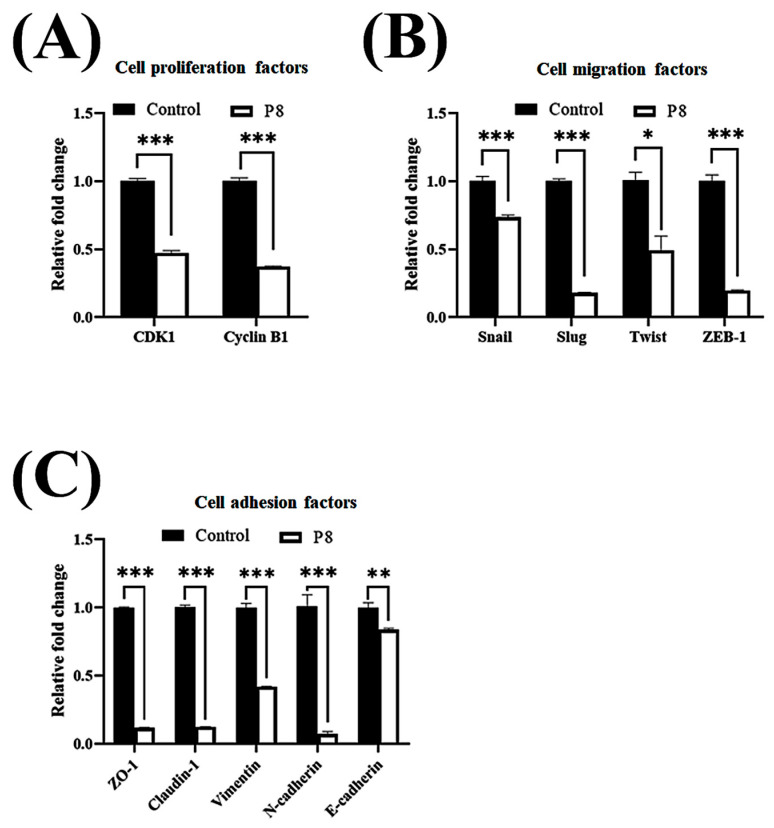
P8-associated changes in the expression of migration and cell adhesion factors in CRC. Quantitative RT-PCR analysis of mRNA levels of genes associated with EMT and cell–cell adhesion in DLD-1 cells treated with P8. DLD-1 cells were treated with 40 µM P8 for 72 h. Levels of expression of (**A**) cyclin B1 and CDK1, (**B**) EMT-associated genes, and (**C**) cell adhesion-associated genes. Relative expression was normalized to that of GAPDH. Data are mean ± SEM from three independent experiments (*n* = 3). * *p* < 0.05, ** *p* < 0.001, *** *p* < 0.001 vs. control.

Smad1 was shown to be highly expressed in CRC tissues, suggesting that Smad1 potentiated CRC cell migration [33]. Smad1 has been implicated in a range of developmental abnormalities and diseases. Its induction by tumor-promoting cytokines, including bone morphogenetic protein 2 (BMP2) and tumor necrosis factor-α (TNF-α), further highlights its role in cell invasion and metastasis [34,35]. The phosphorylated form of Smad1 forms complexes with several types of Smad family proteins, which is important for its ability to regulate the transcription of genes associated with cell invasion and metastasis [36,37,38]. The effects of P8 on Smad1 expression and phosphorylation were therefore analyzed (Figure 3). P8 treatment led to a modest upregulation of Smad1 expression, accompanied by a pronounced reduction in phosphorylation of Smad1 and related complexes, including p-Smad1/5 and p-Smad1/5/8. Phosphorylation of Smad1/5/8 is needed for translocation into the nucleus of DLD-1 cells through the nuclear envelope [39]. Moreover, p-Smad1/5/8 could act as a transcription factor for some genes associated with invasion and metastasis. P8 also markedly reduced the expression of migration factors acting downstream of Smad1, such as Snail, Slug, Twist, and ZEB1 (Figure 3B), as well as significantly reducing the expression of cell adhesion factors, including Zo-1, Claudin-1, vimentin, N-cadherin, E-cadherin, VCAM-1, and ICAM-1 (Figure 3B). Similarly, quantitative RT-PCR showed that P8 down-regulated the expression of mRNAs encoding these proteins (Figure 4). To confirm that DLD-1 cells were susceptible to P8 against DLD-1 cells, the level of expression of the anti-proliferation factors CDK1/Cyclin B1 was measured and found to be significantly decreased by P8 (Figure 4A) [18,19]. Quantitative RT-PCR also showed that P8 negatively altered the levels of expression of cell migration and adhesion factors (Figure 4B,C). Taken together, these findings indicated that P8 could markedly inhibit the phosphorylation of Smad1 and members of the Smad1 family complex, with the reductions in p-Smad and p-Samd1/5/8 leading to the down-regulation of Smad target genes associated with cell migration and adhesion. Moreover, P8 had a negative influence on the levels of expression of focal adhesion factors associated with extracellular matrix adhesion by integrins (Figure S1) [40,41].

### 3.4. Smad1 Dependent Anti-Metastatic Activity of P8

**Figure 5:**
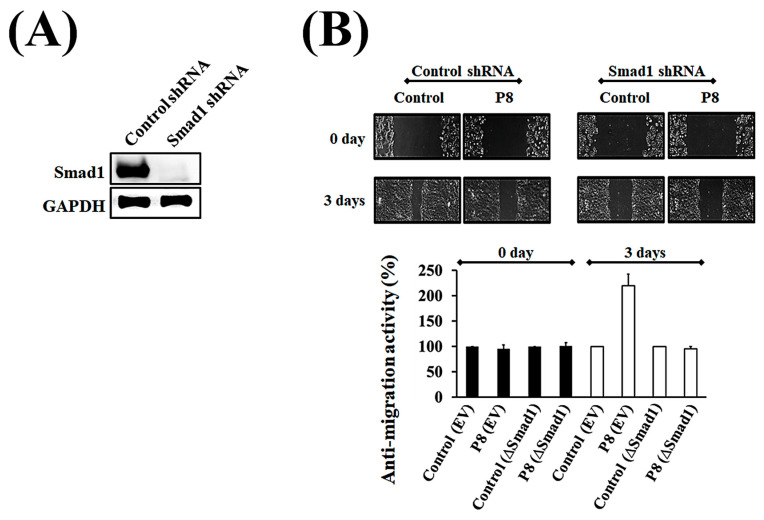
Verification of Smad1 as being associated with the inhibition of migration activity of P8 in DLD-1 cells. To determine whether Smad1 was directly targeted by P8, the P8 sensitivity of ∆Smad1 cells was assessed using the wound healing method. Cells were transfected with Smad1 shRNA or nonspecific shRNA (wild type), and their sensitivity to P8 was compared. (**A**) Assessment of Smad1 knockout by Smad1 shRNA. (**B**) Wound healing assay, showing that ∆Smad1 cells were less sensitive to P8 treatment than wild-type DLD-1 cells. Wound recovery was analyzed using Image J software (Version 1.54p).

To confirm the role of Smad1 in P8-associated anti-metastatic activity, the susceptibility of DLD-1 cells to P8 following knockdown of Smad1 with specific shRNA was evaluated. Transfection of the specific Smad1 shRNA resulted in the successful generation of a ∆Smad1 cell line with a phenotype deficient in Smad1 (Figure 5A). The susceptibility of the ∆Smad1 cell line to P8 was assessed using a wound healing assay, which found that P8 did not alter the migration activity of these cells. By contrast, P8 significantly reduced the migration ability of cells transfected with control shRNA (Figure 5B), indicating that Smad1 is an anti-metastasis target of P8 in CRC.

### 3.5. P8-Induced Changes in Anti-Metastatic Morphology on CRC Surfaces

**Figure 6:**
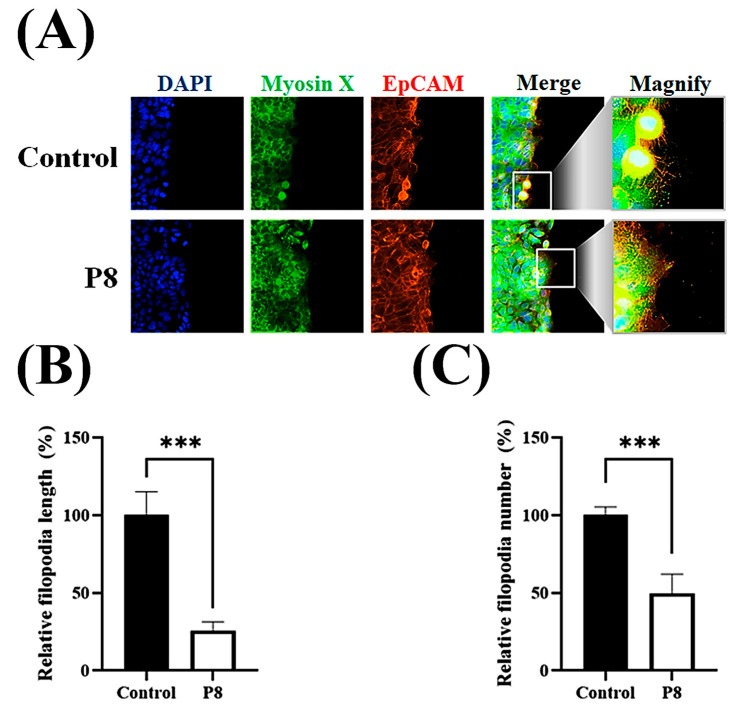
P8-associated suppression of filopodia development on the cell surface. DLD-1 cells were seeded in 96-well plates at 1 × 10^5^ cells per well. After 12 h, a scratch was introduced with a pipette tip, followed by treatment with P8 (40 μM) and incubation for an additional 12 h. Nuclei, myosin X, and EpCMA were visualized by staining with DAPI (blue), FITC (green), and Alexa Fluor 594 (red), respectively. (**A**) ImageXpress^®^ Micro confocal microscopy, showing filopodia formation on cell surfaces after scratching. (**B**,**C**) Analysis of filopodia length (**B**) and number (**C**) with MetaXpress Software v5.3.01 (Molecular Devices). Images were obtained using a 60× objective, and Z-stacks spanned the complete cell clone. Scale bars = 100 μm. Data are mean ± SEM from three independent experiments (*n* = 3). *** *p* < 0.001 vs. control.

**Figure 7:**
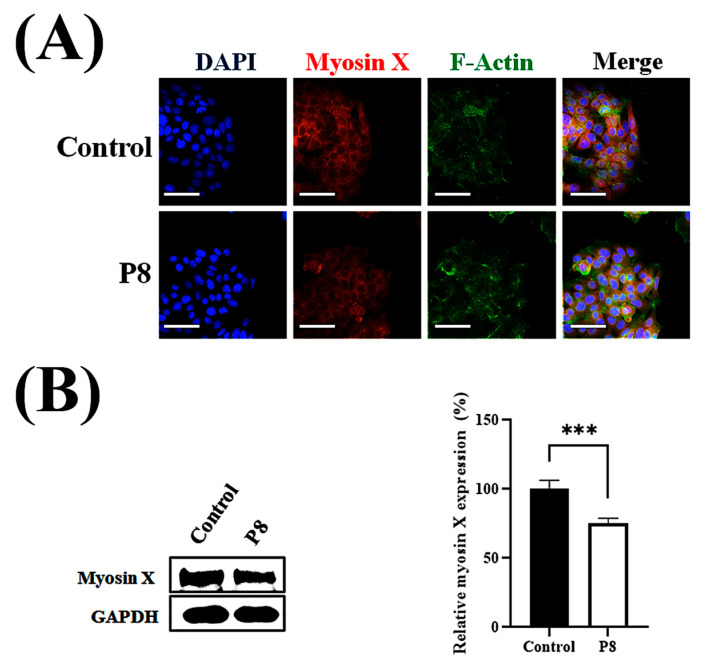
P8-associated reduction in myosin X expression in DLD-1 cells. DLD-1 cells were seeded at 1 × 10^3^ cells per well in 96-well plates. Following a 12 h incubation, P8 (40 μM) was added, and cultures were maintained for 72 h. Nuclei, myosin X, and F-actin were visualized by staining with DAPI (blue), Alexa Fluor 594 (red), and FITC (green), respectively. (**A**) ImageXpress^®^ Micro confocal microscopy, showing cell morphology and myosin X expression, with images analyzed using MetaXpress Software 5.3.01 (Molecular Devices). Images were acquired with a 60× objective, and Z-stacks encompassed the entire cell clone. Scale bars = 100 μm. (**B**) Western blot analysis of myosin X expression in whole-cell lysate, with protein bands quantitatively analyzed using Image J software (Version 1.54p). Data are mean ± SEM from three independent experiments (*n* = 3). *** *p* < 0.001 vs. control.

Cell migration relies on filopodia, which are formed of parallel actin filaments [42,43]. Because filopodia formation should be closely associated with cell migration, the effects of P8 on filopodia number and elongation were investigated (Figure 6). To evaluate the suppression of filopodia formation, filopodia on cell surfaces after scraping were assessed microscopically (Figure 6A). Moreover, the effects of P8 on the expression of the cargo protein myosin X, which is involved in the regulation of filopodia development, were analyzed [44]. Accumulation of myosin X at the filopodia tip was specifically observed during cell migration, with P8 significantly reducing myosin X accumulation at the tips of filopodia (Figure 6A). P8 treatment also reduced relative filopodia length by about ~75% (Figure 6B), as well as significantly reducing the numbers of filopodia (Figure 6C). To determine whether P8 prevents myosin X accumulation at the filopodia tip or reduces myosin X expression in DLD-1 cells, the myosin X expression level was quantitatively analyzed in DLD-1 cells. Myosin X was observed throughout these cells but was slightly reduced following P8 treatment (Figure 7A). Western blot analysis also showed that P8 treatment significantly reduced myosin X expression up to ~25% (Figure 7B).

### 3.6. P8-Induced Changes in Anti-Metastatic Morphology on CRC Spheroid Surfaces

Increasing attention has been directed toward filopodia and filopodium-like protrusions (FLPs) in cancer research [45], since overexpression of proteins such as fascin-1 [46], myosin X [47], and formin [48] enhances metastatic cell motility and invasiveness.

**Figure 8:**
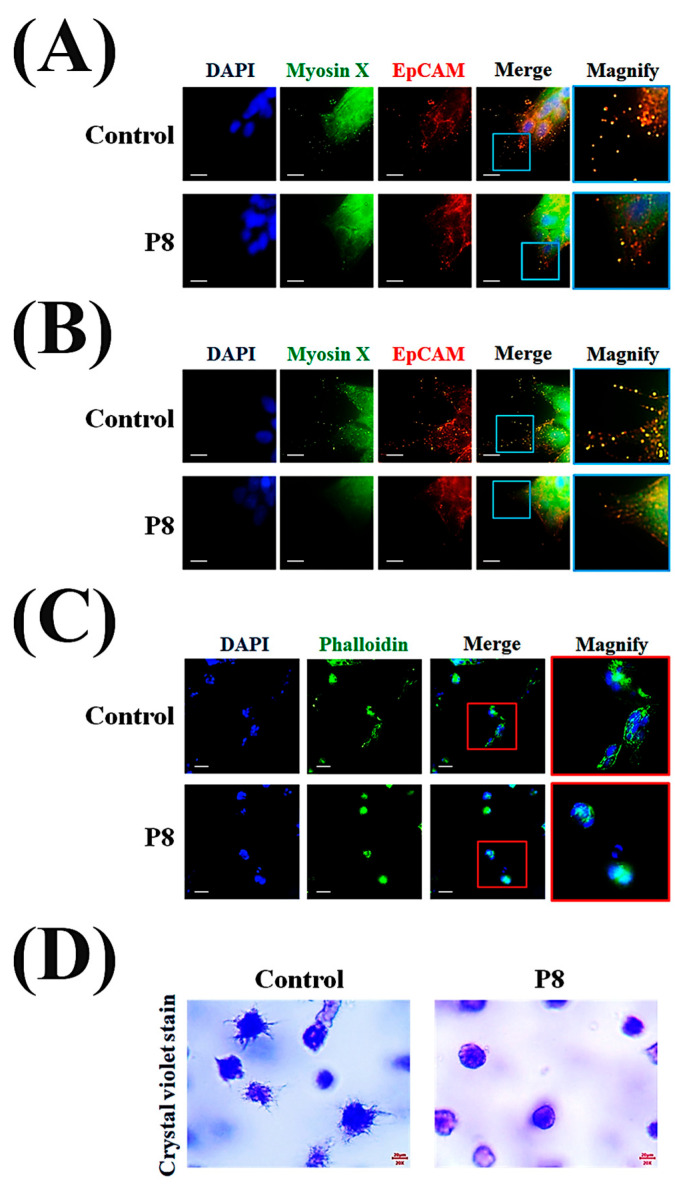
P8-induced morphological changes on spheroid surfaces. DLD-1 cells (2 × 10^4^ cells/well) were incubated with P8 (40 μM) for 8 days using the matrigel 3D culture method. For morphological assessment, cells were imaged with an ImageXpress^®^ Micro confocal microscope and analyzed using MetaXpress Software v5.3.01 (Molecular Devices). A 100× objective was used, and Z-stacks covered the complete cell clone. P8 association suppression of the formation of (**A**) filopodia and (**B**) FLPs was observed on spheroid surfaces. Nuclei, myosin X, and EpCMA were visualized by staining with DAPI (blue), FITC (green), and Alexa Fluor 594 (red), respectively. Scale bars = 20 μm. (**C**) Comparison of early-stage spheroid development by cells in the presence and absence of P8. Five days after seeding, spheroids were visualized by staining of nuclei with DAPI (blue) and F-actin by Phalloidin (green). Scale bars = 100 μm. (**D**) Microscope (Nikon, 200× magnification) examination of the development of early-stage FLPs on spheroid surfaces in the presence and absence of P8. Five days after seeding, spheroids were visualized by staining with crystal violet.

To further assess the metastatic characteristics in tumor cells, the formation of FLPs was analyzed by microscopic evaluation of P8-derived morphological suppression using the MoT 3D culture method [49]. Confocal microscopy showed that P8 treatment not only significantly reduced filopodia formation but also significantly reduced the accumulation of myosin X in filopodia tips (Figure 8A). P8 inhibited FLP formation and branching, as well as reducing the accumulation of myosin X on FLPs (Figure 8B). During early stages, DLD-1 cell spheroids in the absence of P8 showed the development of actin bundles and FLPs, whereas P8-treated spheroids showed restriction of their development (Figure 8C). To determine the P8-associated morphological differences in filopodia or FLPs on spheroid surfaces, these surfaces were stained with crystal violet and evaluated by optical microscopy, with the results showing that P8 significantly inhibited the formation of filopodia and FLPs on spheroid surfaces (Figure 8D).

### 3.7. Anti-Metastatic Effects of P8 in a Mouse Model of CRC Metastasis (Splenic Injection Model)

**Figure 9:**
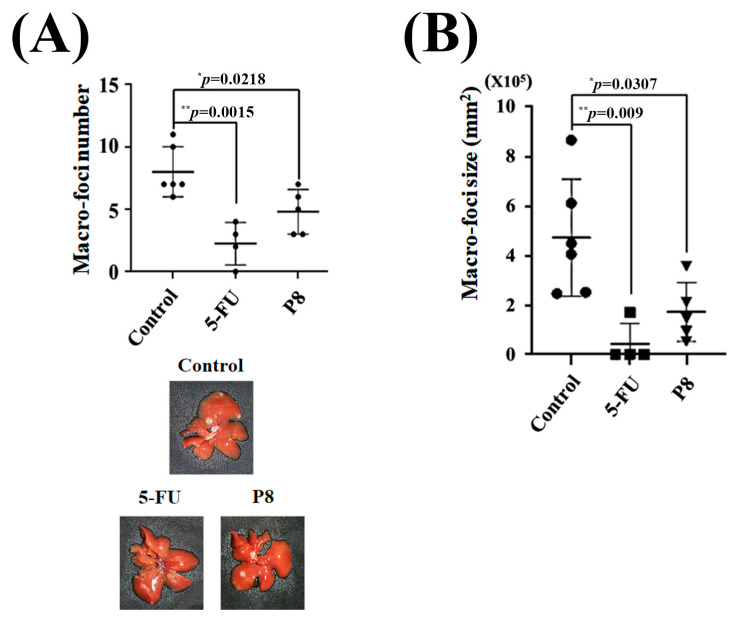
In vivo determination of P8-associated anti-metastatic activity in a mouse model of CRC-associated metastases (splenic injection). Mice received intrasplenic injections of DLD-1 cells and were subsequently treated with P8 (10 mg/kg, four times per week), 5-FU (5 mg/kg, twice per week), or saline as a control. (**A**,**B**) Determination of the (**A**) number and (**B**) size of metastatic foci in the livers of mice. * *p* < 0.05, ** *p* < 0.01 vs. control.

**Figure 10:**
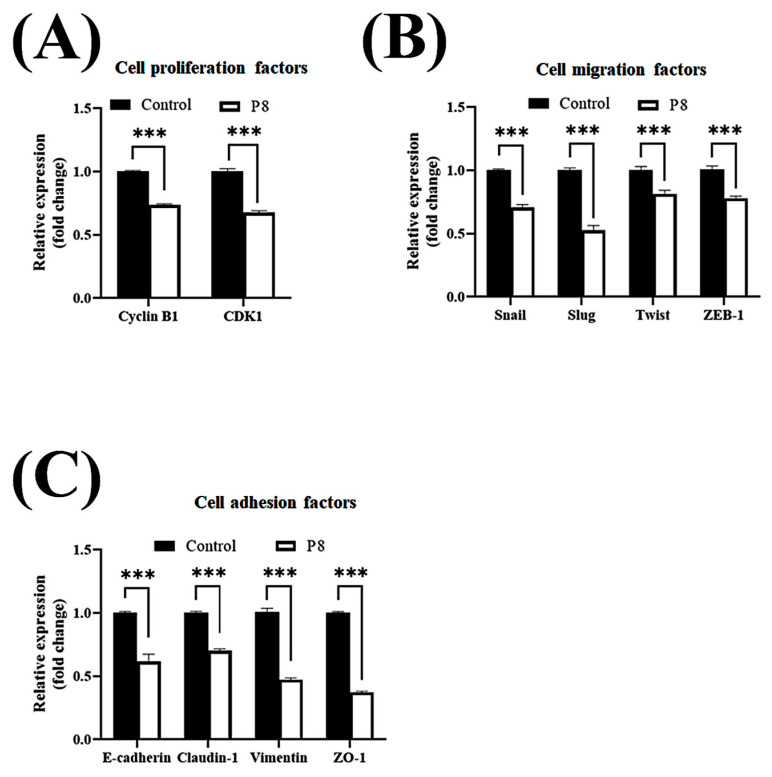
P8-associated changes in the expression of migration and cell adhesion factors in metastatic foci. Quantitative RT-PCR analysis of EMT- and cell–cell adhesion–related gene expression in metastatic foci following P8 treatment. Levels of expression of (**A**) cyclin B1 and CDK1, (**B**) EMT-associated genes, and (**C**) cell adhesion-associated genes. Relative expression was normalized to that of GAPDH. Data are mean ± SEM from three independent experiments (*n* = 3). *** *p* < 0.001 vs. Control.

Taken together, these in vitro results showed that P8 strongly inhibited CRC migration and cell adhesion at both the cellular and molecular levels. The ability of P8 to successfully prevent CRC metastasis in vivo was analyzed using a mouse model of liver metastasis (Figure S2). DLD-1 cells were injected into the spleens of mice, and liver metastases were monitored in the presence or absence of P8 treatment. DLD-1 cells, with or without P8 treatment, formed metastatic foci in the liver, but the number of foci was significantly lower in P8-treated mice than in controls (Figure 9A). To determine whether P8 could affect metastatic growth, the volumes of these metastatic foci were measured, with the volume being significantly lower in P8-treated mice than in controls (Figure 9B). To further investigate the effects of P8 on metastases, the levels of expression of metastasis associated genes related to EMT and cell adhesion were measured in the metastatic foci by quantitative RT-PCR. In Figure 10, P8 was found to strongly inhibit the transcription of each metastatic gene in the metastatic foci, similar to the results in Figure 4. Initially, to confirm that DLD-1 cells derived from metastatic foci were susceptible to P8, the level of expression of the anti-proliferation factors CDK1/Cyclin B1 was measured and found to be decreased by P8 (Figure 10A) [18,19]. Furthermore, P8 negatively altered the levels of expression of cell migration and adhesion factors (Figure 4B,C).

## 4. Discussion

CRC represents the third most frequent cause of cancer-associated death worldwide, with nearly 56% of patients succumbing to the disease [50]. At diagnosis, metastatic disease is detected in roughly 20% of patients [51]. Although early detection of CRC using colonoscopy has reduced CRC mortality rates, early detection has limited effects on all-cause CRC mortality in patients with metastatic cancer at diagnosis [52,53]. Cancer metastasis has become a major concern for both patients and clinicians [54,55], with further developments of treatment methods needed to improve survival rates.

P8, which has been isolated from the probiotic *L. rhamnosus*, has been identified as an anti-cancer protein [18,19], with strong potential as an anti-CRC drug. We have developed a novel anti-CRC therapeutic, consisting of a drug delivery system using *Pediococcus pentosaceus* (*P. pentosaceus*) that secretes P8, with both i.p. injection of P8 protein and recombinant *P. pentosaceus* feeding strongly suppressing tumor growth using a CRC xenograft mouse model [18,28]. P8 was found to inhibit CRC growth through G_2_ cell cycle arrest [18,19,28], as well as showing anti-migration activity in a wound healing assay [19]. However, the MOA of P8 remained unclear.

The present study found that P8 has strong potential as an anti-metastatic drug for CRC. Anti-migration and anti-spheroid formation assays suggested that the anti-metastatic activity of P8 was due to the close association of cell migration and cell–cell adhesion properties with the generation of new metastatic foci in other organs. To understand its anti-metastasis MOA, P8-interacting target proteins in CRC cells were isolated using a P8 pull-down method, a powerful in vitro profiling tool for identifying unknown interacting partners [20,21]. The cellular functions of over 80% of proteins may be manifest only after they form heterogeneous complexes with interacting proteins [56,57]. Profiling the proteins that interact with P8 may be a very effective strategy for determining the MOA of the anti-metastasis activity of P8 in CRC cells [20,21].

Pull-down of putative anti-cancer targets using His-tagged P8 enabled subsequent identification of candidate proteins by LC-MS/MS. P8 was found to interact with Smad1, a transcriptional modulator of the epithelial–mesenchymal transition (EMT) signal pathway activated by BMP2 and TNF-α that plays important roles in cell invasion and metastasis [34,35,58]. Smad1 has been identified as a target of miR-26b-5p in hepatocellular carcinoma metastases and during EMT [59], supporting its involvement in EMT-driven metastatic progression across multiple cancer types. Immunoprecipitation showed that the P8 and Smad1 proteins interacted with each other. Smad proteins (Smad1–9) are central transducers of signals from TGF-β and BMP type I receptors [60,61]. Within the Smad1 pathway, ligand-activated BMP type I receptors directly phosphorylate cytoplasmic Smad1 and Smad1-associated complexes (Smad1/5, Smad1/5/8) [62]. These phosphorylated Smads subsequently translocate and accumulate in the nucleus [63]. Smad1 may play a direct role in transcriptional modulation, acting as a transcription factor [63]. Moreover, Smad1-associated gene regulation has been reported in the metastatic progression of various cancers [33,35,64]. However, the detailed cell signal transduction pathway underlying the negative regulatory link between P8 and Smad1-mediated metastasis remains unclear.

Our findings provide the first evidence that P8 can interfere with the phosphorylation of Smad1 and related complexes (Smad1/5, Smad1/5/8), which is required for their activation, even though Smad1 expression itself was modestly increased. This P8-associated inactivation of Smad1 effectively suppressed the migration of CRC cells by reducing the levels of expression of Snail, Slug, Twist, and ZEB1. Because suppression or reduction in EMT should generally increase the levels of cell–cell adhesion factors [33,65,66], the ability of P8 to alter the expression of cell–cell adhesion factors, including Zo-1, claudin-1, vimentin, N-cadherin, E-cadherin, Vcam-1, and Icam-1, was assessed, with P8 found to reduce the levels of these factors. These findings suggest that we expect that, in addition to the ability to suppress migration, P8 can suppress cell–cell adhesion. Although focal adhesion factors, including Talin1, Tensin2, and vinculin, were significantly reduced, α–actin, Fak, and Paxillin were increased. The P8-associated suppression of cell–cell adhesion factors might correlate with the inhibition of spheroid formation. To determine whether the anti-migration activity of P8 was associated with Smad1, the effect of P8 was tested in cells following knockdown of Smad1. These ∆Smad1 cells were significantly less sensitive to P8 than cells transfected with nonspecific shRNA. Both P8-associated inactivation of Smad1 and Smad1 knockdown yielded CRC cells with a significantly reduced ability to migrate, suggesting that Smad1 is a real target by which P8 suppresses CRC cell migration. Furthermore, Smad signaling is also known to be important for regulating cancer stemness [67]. It is therefore possible that P8 may suppress CRC metastasis not only by inhibiting EMT and migration but also by attenuating stemness.

Migration of existing cancer cells is critical for the formation of new metastatic foci. Filopodia are thin cylindrical extensions of the cell membrane that are filled with long actin filaments organized as a tight bundle with their growing ends pointing toward the direction of protrusion [23]. Filopodia are functionally important for the migration of epithelial cells in wound healing and for the migration of neuronal cells. Additionally, filopodia are thought to be required for probing the ECM environment during migration [68,69]. Elongating filopodia possess specialized tip structures that may play roles in guidance and migration [22].

Filopodia-like structures in vivo, growth factors such as vascular endothelial growth factor and BMPs induce FLPs in cells at the endothelial tip, leading to sprout invasion of the surrounding ECM [70,71]. FLPs are considered essential not only for cancer cell metastasis but also for supporting the survival and proliferation of disseminated carcinoma cells in secondary sites [24]. Although filopodia-derived motility of cancer cells is very important for the progression of metastasis, agents that target filopodia formation and its molecular function can block tumor metastasis [23,43,72,73]. P8-associated morphological changes related to cell migration or adhesion were evaluated by monitoring filopodia formation on the surfaces of CRC cells and spheroids, with results showing that P8 significantly reduced the formations of filopodia and FLPs on both surfaces, thereby significantly reducing the motility of CRC cells.

As a critical regulator of filopodia, myosin X is elevated in breast cancer, and its expression correlates with poor prognosis [74,75]. Overexpression of myosin X enhances filopodia formation [76] and cell invasion [74], whereas loss of its expression impairs invasive capacity both in vitro and in vivo [74,75]. Myosin X likely promotes invasion by trafficking integrin receptors to the tips of filopodia to mediate ECM attachment [74,77]. A feature common to metastatic human cancers suggests that myosin X could drive metastasis of multiple cancer types [78]. Moreover, filopodia formation by cancer stem cells has been associated with the induction of cell migration and tumor metastasis [79]. To better understand P8-associated reductions in filopodia and FLPs in CRC cells and spheroids at the molecular level, the level of myosin X expression or accumulation was investigated in cells and on the tips of filopodia. The significant reduction in myosin X expression in response to P8 treatment indicates that P8-derived CRC morphological changes are directly associated with cell migration, cell–cell adhesion, and spheroid formation. Additionally, P8-associated reductions in cell adhesion molecules may provide further evidence of P8-induced morphological changes in CRC.

Cancer cells that have extravasated into the lungs of mice following tail vein injection have been found to display FLPs. These protrusions harbor tip complexes along their shafts, and their formation is regulated by myosin X, Rho family GTPases such as Cdc42 and Rif, formins, and focal adhesion regulators [24]. The in vivo anti-metastatic activity of P8 was evaluated using a CRC-derived mouse metastasis model. Following splenic injection, CRC cells that had extravasated into the liver formed metastatic foci after migration, adhesion, invasion, and colonization. In this model, P8 significantly reduced the number and size of metastatic foci in the liver. These phenotypes were confirmed at the molecular level by q-PCR, which showed that the expression of factors related to EMT and cell–cell adhesion were suppressed in these metastatic foci. Furthermore, to determine whether P8 accumulates only in metastatic foci but not in normal tissue or accumulates in all liver tissue but more specifically in these foci, P8 distribution in liver tissue was evaluated immunohistochemically, with the results showing that P8 was expressed in all liver tissue but showed greater accumulation in metastatic foci.

## 5. Conclusions

Taken together, these in vitro and in vivo findings indicate that P8 has anti-metastatic properties. To our knowledge, this study is the first to provide evidence about the P8-Smad1-EMT regulatory network or cell adhesion axis in CRC metastasis, suggesting that P8 is a potential new biotherapeutic agent and Smad1 is a potential prognostic factor for CRC metastasis [29]. Moreover, evaluation of the toxicity of the *Pediococcus pentosaceus* SL4-based P8 delivery system (PP-P8) provides valuable data on the safety of P8 and the probiotic-based delivery system, suggesting that P8 may be the first agent originating from a probiotic that can be used in anti-cancer drug development [27].

## Figures and Tables

**Table 1:** Identification of P8-interacting target proteins by LC-MS/MS.

Target Proteins	Symbols	Accession No. (UniProt)	Cellular Functions	Score	Coverage (%)
Mothers against decapentaplegic homolog 1	Smad1	Q15797	Transcriptional modulator activated by BMP (bone morphogenetic proteins) type 1 receptor kinase	32.35	14.84
Peptidyl-prolyl cis-trans isomerase A	PPIA	P62937	Catalyzes the cis-trans isomerization of proline imidic peptide bonds in oligopeptides	27.24	46.67
Sentrin-specific protease 2	SENP2	H7C1A0	Cysteine-type peptidase activity	8.73	17.24
F-box only protein 21	FBXO21	O94952	Ubiquitin-protein transferase activity	141.19	25.64

## Data Availability

The original contributions presented in this study are included in the article/Supplementary Material. Further inquiries can be directed to the corresponding authors.

## Supplementary Materials

The following supporting information can be downloaded at: https://www.mdpi.com/article/10.3390/microorganisms13092175/s1, Figure S1: P8-associated changes in the expression of focal adhesion factors in CRC. q-PCR analysis of mRNAs encoded by genes involved in focal adhesion in DLD-1 cells. Cells were treated with 40 µM P8 for 72 h. Relative expression was normalized to that of GAPDH mRNA. Figure S2: Diagram showing the mouse model of CRC metastasis, involving splenic injection of DLD-1 cells. DLD-1 cells (1 × 10^6^ cfu/mouse) were injected into the spleen or tail vein of NSG immunodeficient mice. The numbers and sizes of metastatic foci in the liver were determined after 7–8 weeks. Based on the numbers and sizes of metastatic foci, the splenic injection method was regarded as better for testing the anti-metastatic activity of P8. Table S1: Primer list for q-PCR analysis.

## Abbreviations

CRC: colorectal cancer; Smad1: mothers against decapentaplegic homolog 1; PP: *Pediococcus pentosaceus*; MOA: mode of action; EMT: epithelial–mesenchymal transition; FLPs: filopodium-like protrusions; BMP: bone morphogenetic protein; TGF: transforming growth factor; MW: molecular weight; IPTG: β-D-1-thiogalacto-pyranoside; GAPDH: glyceraldehyde 3-phosphate dehydrogenase.
