# Supplementary material for: Therapeutic Potential of Probiotic-Derived P8 Protein as an Anti-Metastatic Agent in Colorectal Cancer
Source: Microorganisms. 2025 Sep 17;13(9):2175. doi: 10.3390/microorganisms13092175 (PMC12472984; doi:10.3390/microorganisms13092175)
Supplement: Supplementary file 1 [file microorganisms-13-02175-s001.zip › Table S1. Primer list for q-PCR analysis.pdf]

Table S1. Primer list for q-PCR analysis.

Target genes	Symbols	Primer names	Primer sequences
**Glyceraldehyde-3-phosphate dehydrogenase**	**GAPDH**	F	CCT TCA TTGACC TCA ACT ACA TGG
		R	GTC TTCTGG GTG GCA GTG ATG G
**G2/mitotic-specific cyclin-B1**	**Cyclin B1**	F	AAGAGCTTTAAACTTTGGTCTGGG
		R	CTTTGTAAGTCCTTGATTTACCATG
**Cyclin-dependent kinase 1**	**CDK1**	F	TGGATCTGAAGAAATACTTGGATTCTA
		R	CAATCCCCTGTAGGATTTGG
**Vimentin**	**Vimentin**	F	ACACCCTGCAATCTTTCAGACA
		R	GATTCCACTTTGCGTTCAAGGT
**Claudin1**	**Claudin1**	F	TGCGATATTTCTTCTTGCAGGT
		R	TTCGTACCTGGCATTGACTGG
**Cadherin-1**	**E-cadherin**	F	AAGGAGGCGGAGAAGAGGAC
		R	CGTCGTTACGAGTCACTTCAGG
**Zinc finger protein SNAI1**	**Snail**	F	TTCCAGCAGCCCTACGACCAG
		R	CGGACTCTTGGTGCTTGTGGA
**Zinc finger protein SNAI2**	**Slug**	F	TTC GGA CCC ACA CAT TAC CT
		R	GCA GTG AGG GCA AGA AAA AG
**Zinc finger E-box-binding homeobox 1**	**ZEB1**	F	AGCAGTGAAAGAGAAGGGAATGC
		R	GGTCCTCTTCAGGTGCCTCAG
**Tight junction protein ZO-1**	**ZO1**	F	TAT TAT GGC ACA TCA GCA CG
		R	TGG GCA AAC AGA CCA AGC
**Twist-related protein 1**	**Twist**	F	GGCTCAGCTACGCCTTCTC
		R	TCCTTCTCTGGAAACAATGACA
