# Supplementary figures and images for: Therapeutic Potential of Probiotic-Derived P8 Protein as an Anti-Metastatic Agent in Colorectal Cancer
Source: Microorganisms. 2025 Sep 17;13(9):2175. doi: 10.3390/microorganisms13092175 (PMC12472984; doi:10.3390/microorganisms13092175)

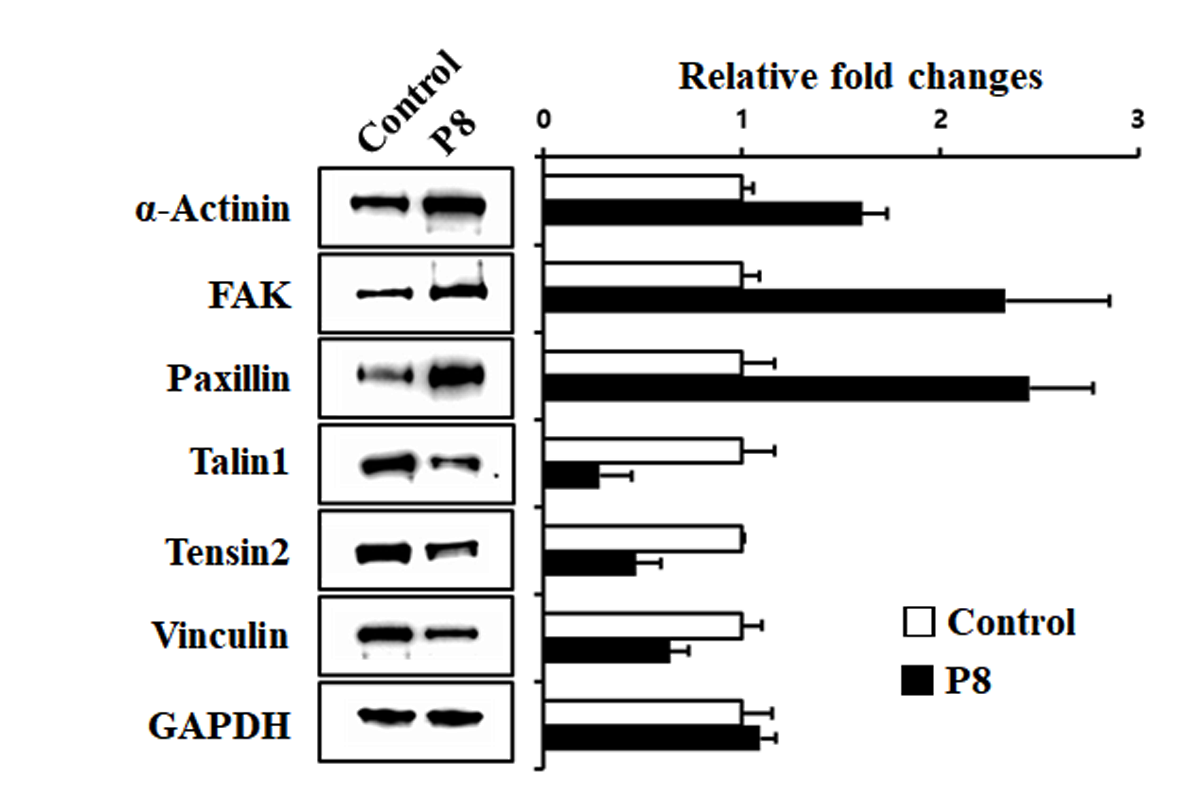

Supplement: Supplementary file 1 [file microorganisms-13-02175-s001.zip › Supplementary Figure S1.tif]

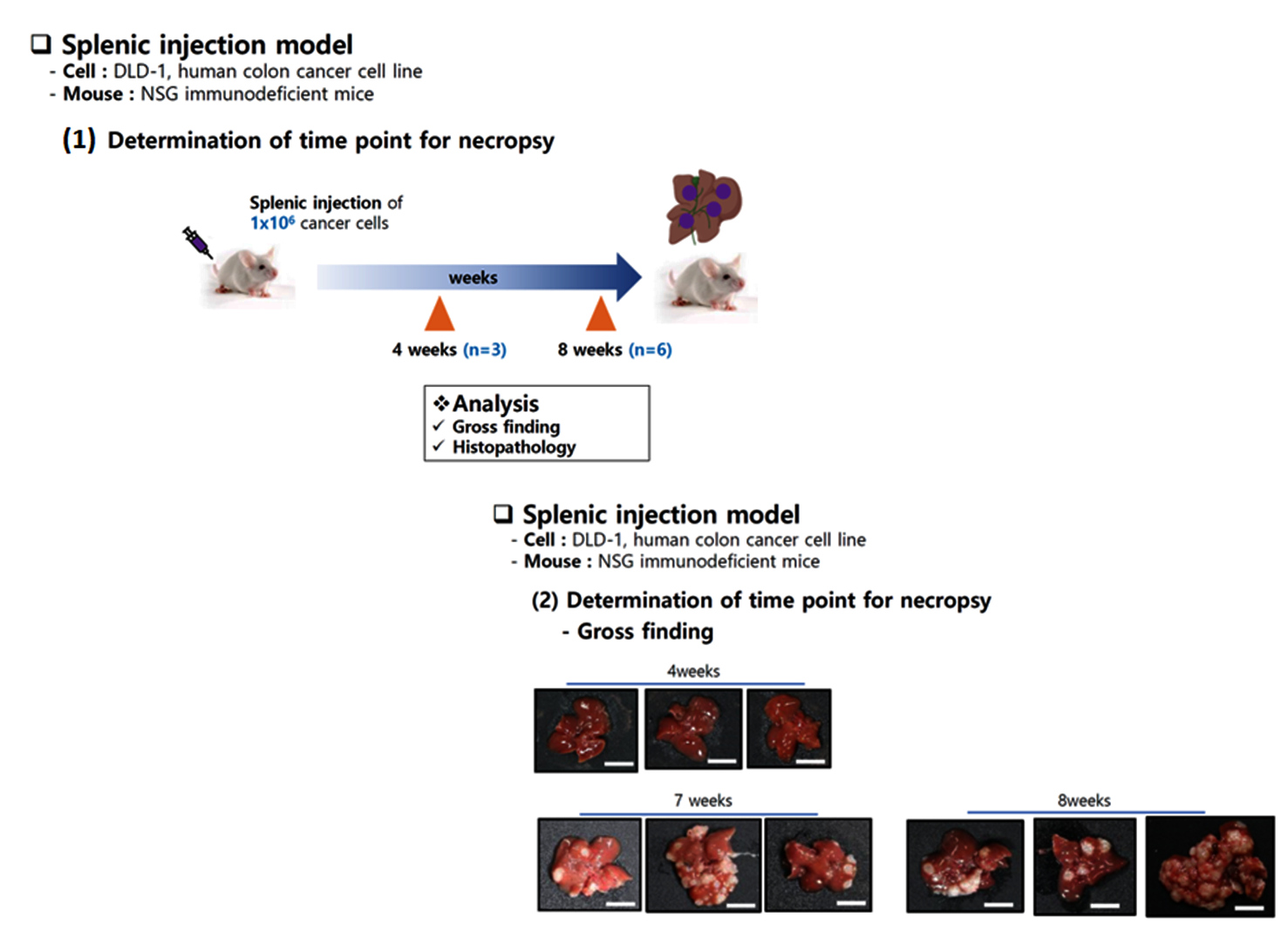

Supplement: Supplementary file 1 [file microorganisms-13-02175-s001.zip › Supplementary Figure S2.tif]
